# Organic Fertilizer and Amendment Improve Physical Properties of Albic Soil Under Crop Rotations

**DOI:** 10.3390/plants15101554

**Published:** 2026-05-19

**Authors:** Yue Zhao, Yu Zheng, Yuchao Song, Xiaoyu Hao, Jinghong Ji, Shuangquan Liu, Lingli Wang, Xingzhu Ma

**Affiliations:** 1 Heilongjiang Academy of Agricultural Sciences/Nangang, Soil Quality, Observation and Research Station, Key Laboratory of Black Soil Conservation and Utilization, Ministry of Agriculture and Rural Affairs, Harbin 150086, China; zhaoyue2108@163.com (Y.Z.); annadian@163.com (Y.Z.); xiaoyuhao1981@sina.com (X.H.); jinghong_98@163.com (J.J.); shuangquanliu@126.com (S.L.); 2 Institute of Applied Ecology, Chinese Academy of Sciences, Shenyang 110016, China; songyuchao@iae.ac.cn; 3 Shenyang Zhongke New Fertilizer Co., Ltd., Shenyang 110016, China

**Keywords:** soil amendment, physical properties, size distribution of aggregates, maize yield

## Abstract

Albic soil is a typical low-yield and problematic soil in Northeast China, which severely restricts crop growth and yield. Through a three-year field experiment in the albic soil of China, soil three-phase ratio, soil bulk density (soil BD), soil total porosity (soil TP), size distribution and water stability of soil aggregates, and changes in maize yield under crop rotations were studied. The following three treatments were established: conventional fertilization (T0), conventional fertilization + soil amendment (T1), and conventional fertilization + soil amendment + bio-organic fertilizer (T2). The results indicated that, compared with T0, the soil three-phase ratio deviation value (R) of T1 and T2 decreased by 23.69–74.94%, and the generalized soil structure index (GSSI) increased by 4.75–15.41% in soil layers of 0–20 cm and 20–40 cm. Soil BD and soil TP changed significantly in the soil layer of 20–40 cm under treatment of T2 (decreased 12.82% and increased 18.31%, respectively). Content of aggregates >0.25 mm (R_0.25_) increased significantly in the soil layer of 0–20 cm, with increases of 13.71% and 23.21% under treatments of T1 and T2. The mean weight diameter (MWD) and geometric mean diameter (GMD) increased significantly under treatment of T2. Compared with T0, maize yield increased 13.45% and 18.85% under treatments of T1 and T2. Correlation analysis showed that maize yield was significantly correlated with soil physical indexes and aggregate stability. In summary, the combined application of soil amendments and bio-organic fertilizer is not only important for albic soil improvement, but also crucial to stabilize the crop production capacity in albic soil regions.

## 1. Introduction

Albic soil, which is one of the main soil types in Northeast of China, is mainly distributed in the Sanjiang Plain and accounts for about 1/4 of the area of cultivated land in this region, most of which are medium- and low-yield fields [1]. Albic soil has the albic horizon in the 20–40 cm, which is compact and hard; it affects water movement and nutrient transport and also restricts the downward growth of crop roots, seriously hinders crop growth and limits the increase in crop yield [2]. Therefore, improvement of albic horizon is of great significance for enhancing grain production capacity.

Soil physical properties and size distribution are comprehensive indicators that characterize the exchange, transport and supply status of water–fertilizer–air–heat in soil and play an important role in regulating soil fertility and crop growth [3]. Physical and chemical improvement are important ways for enhancing structure of albic soil. Firstly, physical improvement is realized by mechanical tillage to break the albic horizon and ameliorate soil physical structure, thereby increasing the permeability of the albic horizon [4,5,6]. Ma et al. showed that subsoiling tillage improves soil structure significantly and makes it closer to the ideal state [7]. Straw returning combined with organic materials can reduce the hardness of albic soil, improve the soil fertility, and increase the yield of soybean [8,9]. Crop yields increase about 30%, 10% and 7% in albic soil, black soil and chernozem respectively under treatments of fertilization in subsoil [10,11,12]. Secondly, chemical improvement promotes the formation of soil aggregates, optimizes soil structure, and further improves soil fertility by adding exogenous organic and inorganic materials [13]. The application of soil amendments such as montmorillonite regulates soil physical and chemical properties effectively, such as reducing soil BD and increasing soil TP, which are beneficial for crop growth [14,15]. The application of organic fertilizer reduces soil BD effectively [16], promotes the transformation of micro-aggregates in clay into larger-sized aggregates, and optimizes the weight ratio of aggregates of each particle size [17]. Ren et al. pointed out that the application of bio-organic fertilizers increases the proportion of large aggregates and the MWD of soil aggregates significantly [18]. Compared with conventional fertilization, the application of bio-organic fertilizer combined with shell powder can improve the properties of protected vegetable soil and reduce soil BD significantly [19]. Other studies have shown that soil amendments can enhance soil permeability and create more favorable environment for root and crop growth, ultimately leading to an increase in production [20,21,22]. Studies of soil improvement focused on single physical or chemical improvement measures mostly; those measures have achieved certain results regarding soil physical properties improvement and increasing of crop yield, while, aiming at the typical characteristics of albic soil, such as the hard and compact albic horizon and severe structural dysfunction, the single treatment or technology has difficulty realizing the synergistic improvement of profile obstacle reduction and soil fertility enhances fundamentally. At present, systematic research on the combined mode of “applying soil amendments in the subsoil and bio-organic fertilizer in the topsoil” and its influence on soil physical properties and crop yield in albic soil are still scarce, and the related mechanisms remain unclear.

Can the combined measures simultaneously improve size distribution of soil aggregates and reduce soil compaction, thereby increasing crop yield more significantly than the single measures? Based on this scientific hypothesis, this study took low-yield albic soil as the research object and adopted comprehensive physical, chemical, and biological improvement and fertilization measures. It systematically analyzed the changes in soil physical properties and maize yield, revealing the synergistic mechanism of “inorganic-organic” fertilization measures. Results are expected to provide a theoretical basis and technical support for the improvement of albic soil and enhancement of crop yield in this region.

## 2. Results

### 2.1. Changes in Soil Physical Properties

#### 2.1.1. Soil Three-Phase Ratio

The soil three-phase ratio under different fertilization is shown in Table 1. Results showed that the solid and liquid phase ratios in the soil layer of 20–40 cm were higher than those in 0–20 cm, and the gas phase ratio was lower than that of the topsoil. Compared with T0, the soil liquid phase ratio under treatments of T1 and T2 increased significantly in the soil layer of 0–20 cm, about 18.70% and 27.25%; the gas phase ratio decreased by 7.61% and 9.59%, respectively. The soil solid phase ratio of 20–40 cm soil layer under treatments of T1 and T2 decreased significantly (19.05% and 15.19%, respectively), and the gas phase ratio increased by 103.54% and 63.39%, respectively. Compared with T0 and T1, the liquid phase ratio of T2 increased significantly, with an average increase of 9.97%.

**Figure 1:**
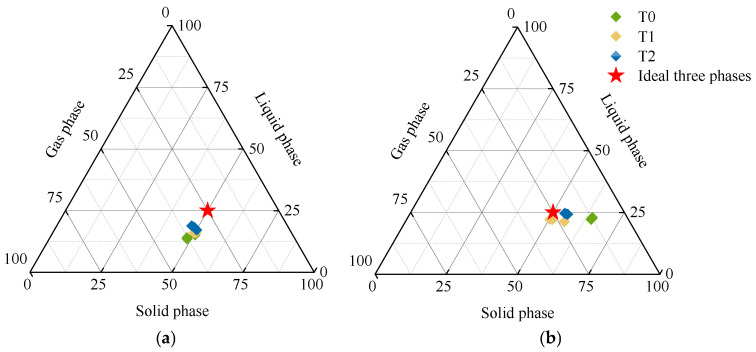
Soil three-phase ratio under different fertilization treatments. (**a**) 0–20 cm soil layer; (**b**) 20–40 cm soil layer.

As the soil layer deepened, the GSSI of T0 decreased, and the soil three-phase deviation value (R) increased, while T1 and T2 were opposite (GSSI increases, R decreases) (Table 2). Compared with 0–20 cm soil layer, the GSSI of T1 and T2 increased by 5.06% and 2.87% in 20–40 cm soil layer, respectively, and the R decreased by 60.02% and 38.88%, respectively. Compared with T0, the GSSI of T1 and T2 in the 0–20 cm soil layer increased significantly (increased by 4.75% and 6.22%, respectively), and the R decreased significantly (23.69% and 31.94%, respectively). Meanwhile, in the 20–40 cm soil layer, the GSSI of T1 and T2 increased significantly, increasing by 15.41% and 14.60%, respectively; the R decreased significantly (decreased by 74.94% and 65.83%, respectively). T1 and T2 could adjust the GSSI and R; the soil three-phase ratio of the soil was closer to the ideal state, especially in the soil layer of 20–40 cm (Figure 1).

#### 2.1.2. Soil BD and Soil TP

**Figure 2:**
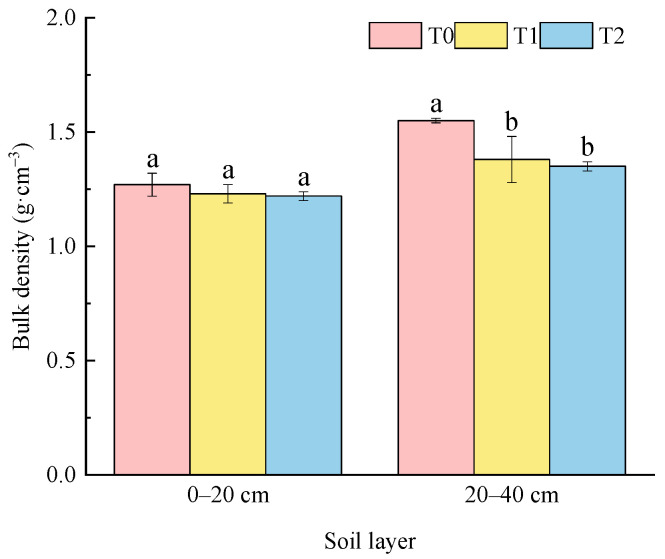
Soil bulk density under different fertilization treatments. Note: different lowercase letters indicate significant differences between treatments (*p* < 0.05).

Figure 2 showed the changes in soil BD under different fertilization in different soil layers. The trend showed that soil BD increased gradually with the increase in soil depth. In soil layers of 0–20 cm and 20–40 cm, soil BD under different fertilization followed by T2 < T1 < T0, and they changed significantly in the lower soil layer (20–40 cm). Soil BD ranged from 1.22 g·cm^−3^ to 1.27 g·cm^−3^ in the layer of 0–20 cm, with an average value of 1.24 g·cm^−3^, and no significant difference was observed among all treatments. Soil BD varied from 1.35 g·cm^−3^ to 1.55 g·cm^−3^, with an average value of 1.43 g·cm^−3^ in the soil layer of 20–40 cm. Compared with T0, T1 and T2 significantly reduced soil BD by 10.60% and 12.82%, respectively.

**Figure 3:**
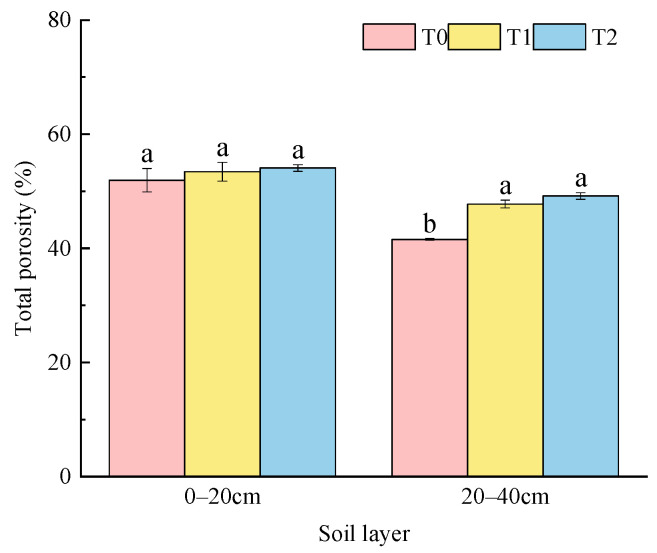
Soil total porosity under different fertilization treatments. Note: different lowercase letters indicate significant differences between treatments (*p* < 0.05).

The change in soil TP was opposite to that of soil BD (Figure 3); soil TP under different fertilization followed the order of T2 > T1 > T0 in soil layers of 0–20 cm and 20–40 cm. As the soil layer deepens, soil TP decreases. Under T0, T1 and T2, soil TP in the 20–40 cm layer was 19.95%, 10.58% and 9.08% lower than that in 0–20 cm, respectively; soil TP ranged from 51.93% to 54.09%, with an average value of 53.14%, and there was no significant difference among treatments in the 0–20 cm layer, while the difference was significant in the 20–40 cm layer—it varied from 41.57% to 49.18%, with an average value of 46.17%. Compared with T0, T1 and T2 significantly increased soil TP by 14.89% and 18.31%, respectively.

### 2.2. Size Distribution and Water Stability of Soil Aggregates

#### 2.2.1. Soil Aggregate Size Distribution

**Figure 4:**
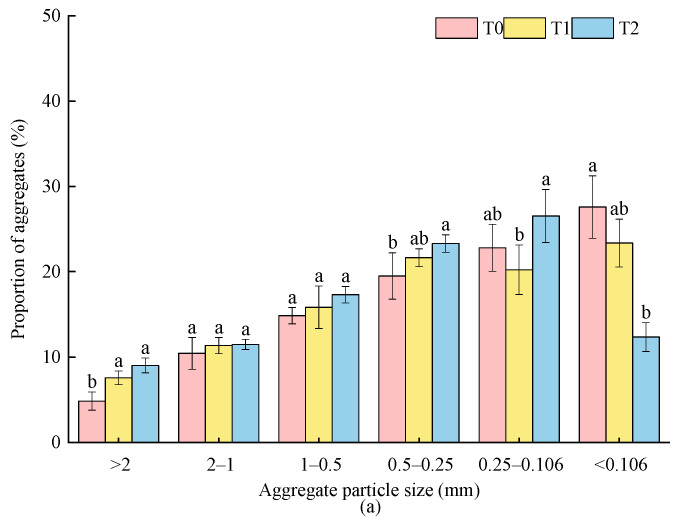

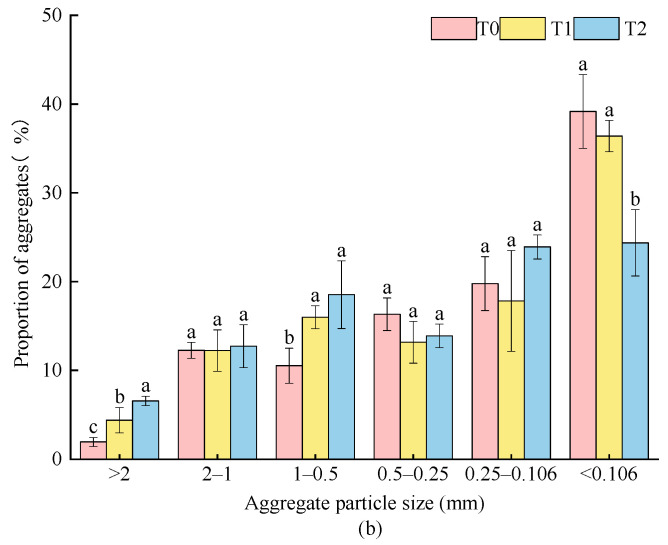
The size distribution of soil aggregates under different treatments. (**a**) 0–20 cm soil layer; (**b)** 20–40 cm soil layer. Note: different lowercase letters indicate significant differences between treatments (*p* < 0.05).

The size distribution of soil aggregates under different fertilization is shown in Figure 4. With the increase in soil depth, the proportion of aggregates <0.106 mm increased. In the 0–20 cm soil layer, the proportion of >0.25 mm aggregates in T1 and T2 increased; among them, the proportions of aggregates >2 mm and 0.5–0.25 mm in T2 were significantly increased by 86.74% (from 4.83% in T0 to 9.01% in T2) and 19.52% (from 19.50% in T0 to 23.31% in T2), respectively. Meanwhile, the proportion of aggregates <0.106 mm was from 27.59% in T0 to 12.35% in T2, a significant decrease of 55.25%. The proportions of aggregates of all particle sizes >0.106 mm in T2 were higher than those in T1, a significant difference only for 0.25–0.106 mm aggregates (increased by 31.21%). In the 20–40 cm soil layer, compared with T0, the proportions of aggregates >2 mm under treatments of T1 and T2 were significantly increased by 124.57% and 236.52%, respectively; the proportions of 1–0.5 mm aggregates were significantly increased by 51.77% and 75.89%, respectively; and the proportion of aggregates <0.106 mm in T2 was decreased (by 37.8%) significantly. Compared with T1, T2 increased proportion of aggregates >2 mm (by 49.85%) significantly and decreased proportion of aggregates <0.106 mm (by 33.05%) significantly.

#### 2.2.2. Stability of Soil Aggregates

Analysis of soil aggregate stability (Table 3) showed that, compared with the 0–20 cm soil layer, the R_0.25_, MWD and GMD of soil in the 20–40 cm soil layer all decreased under different treatments. R_0.25_, MWD and GMD all followed the order of T2 > T1 > T0 in two soil layers. In the 0–20 cm soil layer, compared with T0, R_0.25_ and MWD of T1 and T2 were significantly increased by 13.71% and 23.21% (R_0.25_), 20.92% and 34.29% (MWD); GMD showed an increasing trend, and it was increased by 48.63% significantly. Treatment of T2 increased MWD and GWD by 51.01% and 54.24% in soil layer of 20–40 cm.

### 2.3. Maize Yield

Maize yields under different fertilization are shown in Table 4. Maize yields under different treatments followed the order T2 > T1 > T0; the highest maize yield of T2 was observed, with yield as high as 11,434.00 kg·ha^−1^. The maize yields of T1 and T2 were both higher than T0 significantly, which increased by 13.45% and 18.85%, respectively. The maize yield of T2 was 4.76% higher than T1. Therefore, the application of soil amendment influenced the yield, increasing significantly.

### 2.4. Correlation Between Soil Physical Properties and Maize Yield

**Figure 5:**
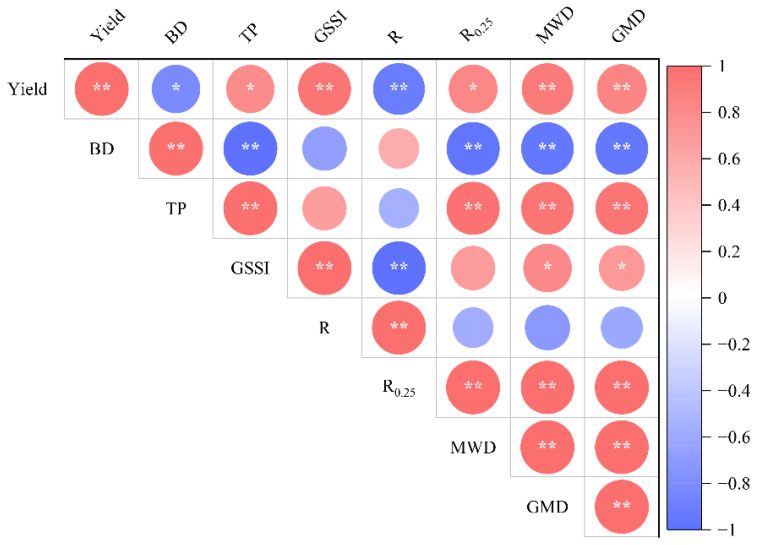
Correlation analysis between soil physical properties, size distribution and water stability of soil aggregates and maize yield. Note: yield, BD, TP, GSSI, R, R_0.25_, MWD and GMD refer to yield, soil bulk density, soil total porosity, generalized soil structure index, soil three-phase deviation value, >0.25 mm aggregate ratio, mean weight diameter and geometric mean diameter; * indicates *p* < 0.05, ** indicates *p* < 0.01.

Analysis of correlation between maize yield and soil physical indexes is shown in Figure 5. The results showed that maize yield was positively and significantly correlated with GSSI, MWD and GMD; the correlation coefficients were 0.77, 0.71 and 0.63, respectively, which was also positively and significantly correlated with soil TP and R_0.25_; the correlation coefficients were 0.48 and 0.58, respectively, while maize yield was negatively and significantly correlated with soil BD (r = −0.50) and negatively and significantly correlated with R (r = −0.71). Soil BD and GSSI were negatively and significantly correlated with soil TP (r = −1.00) and GSSI (r = −0.98), respectively. R_0.25_, MWD and GMD all showed negative and significant correlations with soil BD, and the correlation coefficients were −0.77, −0.74 and −0.70, respectively; and there was also a significant and positive correlation with soil TP (coefficients were 0.76, 0.73 and 0.70, respectively).

## 3. Discussion

### 3.1. Changes in Albic Soil Physical Properties Under Different Fertilization

Application of soil amendment could improve the structure of topsoil and regulate the three-phase ratio of soil in 0–40 cm layer. In this study, combined application of soil amendment and bio-organic fertilizer could promote the soil three-phase ratio closer to the ideal state of 2:1:1 (Table 1). The application of soil amendment and organic materials could break the compact structure of the albic horizon, increase soil permeability, and optimize the soil three-phase ratio, which was consistent with those results of Wang et al. [14]. However, other studies have shown that excessive application of organic materials deviated the soil three-phase ratio from the ideal value, mainly because large amounts of organic materials led to rapid water evaporation from soil and increased soil gas content, thus making the soil too loose and deteriorating the structure [23,24]. The specific effect of different measures on improving soil three-phase ratio needs to be further verified. Compared with the conventional treatment, application of soil amendment alone and its combination with bio-organic fertilizer both increased the GSSI and decreased the R (Table 2 and Figure 1). It is worth noting that the single application of soil amendments exhibited better improvement effects on soil GSSI and R than the treatment combined with bio-organic fertilizer in the soil layer of 20–40 cm. A possible reason was that the organic fertilizer applied in the 0–20 cm layer was prone to downward leaching and migrated to the 20–40 cm layer with soil water movement. Fine organic colloids blocked soil aeration pores, decreased the gas phase proportion of subsoil and caused water stagnation [25]. In addition, the improvement effect in 20–40 cm soil layer was better than that in 0–20 cm soil layer (Table 2 and Figure 1). This was consistent with the result of Gao et al., which was related to the application of soil amendment, and its combination with bio-organic fertilizer could optimize the soil three-phase ratio and improve soil structure stability [8]. Studies have pointed out that mixed application of soil amendments such as shell powder, straw biochar and slag with bio-organic fertilizer had significant effects on improving soil BD and soil TP [19,26,27]. In this study, application of soil amendment reduced soil BD and increased soil TP in 0–40 cm soil layer, and there was a significant effect under the treatment of T2 (Figure 2 and Figure 3), which was consistent with previous studies [19,26,27]. The reason might be related to the components in the soil amendment which absorbed water and swell, then improved the soil pore structure and reduced soil compaction through physical filling. In addition, the decomposition of bio-organic fertilizer formed a porous structure and increased the pore space; the combination of the two could form a stable pore structure to achieve better improvement effect [28]. Numerous studies pointed out that reasonable mechanical disturbance of soil is also an important factor for improving soil physical properties [4,12,29,30].

### 3.2. Effects of Different Fertilization on Size Distribution and Water Stability of Soil Aggregates of Albic Soil

Soil aggregates are the basic units of soil structure, which can be divided into macroaggregates (>0.25 mm) and microaggregates (<0.25 mm) according to particle size. The higher the R_0.25_, the stronger the stability and erosion resistance of soil structure. Its stability was usually characterized by parameters such as MWD and GMD; the larger the value, the more stable the water stability of soil aggregates [31,32,33]. In this study, compared with conventional fertilization, soil amendment increased the content of large-sized soil aggregates, promoted the transformation of microaggregates to macroaggregates, and improved the R_0.25_, MWD and GMD. Under treatment of bio-organic fertilizer, the aggregate stability indexes increased significantly, and the size distribution and water stability of soil aggregates was more stable (Figure 4 and Table 3), which was consistent with previous studies [14,34]. On the one hand, mechanical operation broke the hard and compact albic horizon of albic soil through external force, relieved the compaction restriction of the original soil, and then drove the improvement of size distribution and water stability of soil aggregates and the enhancement of stability [29]. On the other hand, it might be related to the raw materials of the amendment applied in this experiment. Modified bentonite, one of the raw materials, had a high specific surface area and cementing property, which could promote the transformation of soil microaggregates to macroaggregates (>0.25 mm) through flocculation between small soil particles [35]. Another raw material was modified humic acid, which acted as an organic cementing agent and could be combined with cations in soil through organic functional groups to form the core cementing substance of aggregates [36]. The amendment with calcium oxide as raw material used Ca^2+^ as a “bridge” to connect negatively charged soil colloids to form a stable size distribution and water stability of soil aggregates [37]. Meanwhile, during the decomposition process, bio-organic fertilizer could produce substances such as organic acids, which promoted the aggregation of soil particles; it also formed a physical scaffold through the interweaving of fungal hyphae, wrapped and fixed soil particles into aggregates, significantly reduced the proportion of micro-aggregates, and optimized the aggregate distribution structure, thereby improving the stability of soil aggregates [38,39].

### 3.3. Changes in Maize Yield and Its Factors in Albic Soil Area

Both soil amendment application alone and its combined application with bio-organic fertilizer could increase maize yield, and the effect of increasing yield was more significant under the combined application of bio-organic fertilizer (Table 4). One of the reasons might be that bio-organic fertilizer is rich in organic matter and beneficial microorganisms; reasonable application could reduce soil BD, promote nutrient cycling, improve soil fertility, and thus increase crop yield [40,41,42,43]. Another possible reason was the intervention of machinery reduced the growth stress caused by soil compaction, promoted the coordinated development of vegetative and reproductive growth of crops, and finally achieved yield increase [44]. Correlation analysis showed that soil physical indexes and aggregate indexes were significantly or extremely significantly correlated with maize yield (Figure 5). Overall, the combined application of soil amendments and bio-organic fertilizers improved soil physical structure and enhanced soil aeration, water permeability, as well as soil water and nutrient retention capacity [8,34]. A well-aggregated soil structure not only provided favorable physical space for crop root downward penetration and lateral extension but also increased root activity by retaining soil organic carbon and nutrient substrates. Developed root systems in turn further promoted the formation and stability of soil aggregates through physical entanglement and the cementation of root exudates [34]. In this process, the absorption and utilization efficiency of soil water and nutrients by roots was greatly improved, which promoted the growth of above-ground plant parts and the accumulation of photosynthates, ultimately increasing maize yield [45]. This study did not set up a separate bio-organic fertilizer treatment to explore the individual effects of amendments and organic fertilizers. Instead, it mainly focused on analyzing the comprehensive effects of combined organic and inorganic fertilization, amendment application, and mechanical practices on the soil–crop system. Overall, the integrated measures exhibited a positive regulatory effect.

## 4. Materials and Methods

### 4.1. Study Site

The experiment was conducted at the Scientific Research Station of 852 Farm Management Zone (46°17′ N, 132°44′ E) of Shuangyashan City, Heilongjiang Province, China. This experiment was conducted from 2023 to 2025, following a maize–soybean rotation system with one crop per year. This area belongs to the sub-frigid monsoon climate zone, with an average annual temperature of 2.8–3.2 °C and an annual precipitation of 458 mm. The soil type is upland of albic soil, with the albic horizon distributed in the soil layer of 20–40 cm. At the start of the experiment, the contents of soil organic matter, total phosphorus, and total nitrogen in the 0–20 cm soil layer were 34.10, 0.59 and 1.55 g·kg^−1^, respectively; available phosphorus and alkali-hydrolyzable nitrogen were 9.78 and 197.05 mg·kg^−1^, respectively; soil pH was 5.84 and soil bulk density was 1.28 g·cm^−3^.

### 4.2. Experimental Design

We made use of a field experiment, and the following three treatments were set up: conventional fertilization (T0), conventional fertilization + soil amendment (T1), conventional fertilization + soil amendment + bio-organic fertilizer (T2). Each treatment had 3 replications arranged in a randomized block design, and the area of each plot was about 520 m^2^ (ridge width 1.3 m × ridge length 25 m × 16 ridges). The conventional fertilization treatment used formula fertilizer (nutrient content of N-P_2_O_5_-K_2_O was 26-10-12). The soil amendment was a mixture of modified bentonite, modified humic acid and calcium oxide, which were compounded at a weight ratio of 1:1:0.5. Bentonite has high water absorption and swelling capacity; humic acid acts as an organic cementing agent; and calcium oxide provides Ca^2+^ as a bridging cation. The bio-organic fertilizer was produced by Liaoning Xingnuo Agricultural Development Technology Co., Ltd., which takes agricultural and livestock by-products as the main raw materials and inoculates functional compound microbial agents, containing effective viable bacteria ≥0.20 billion per gram and organic matter ≥40%.

The bio-organic fertilizer was spread on the surface soil before sowing, then deep plowed to 20 cm and fully mixed with the topsoil layer mechanically; the formula fertilizer was applied during period of sowing; and the soil amendment was applied to the soil layer of 20–40 cm, using a self-developed subsoil fertilization plow after the autumn harvest of the previous year (20 October 2024). The plow adopted an upper-turning and lower-loosening operation mode, with upper turning of 20–25 cm and lower loosening of 20 cm, and with a total deep tillage depth of 40–45 cm. The application rates of fertilizers and amendment are shown in Table 5.

The experimental crops were planted on 15 May 2025, and harvested on 22 September 2025. The tested crop was maize (*Zea mays* L.) and the variety was Songyu 438, an early-maturing variety requiring an accumulated temperature of 2350–2450 °C above 10 °C during the whole growth period, with a planting density of 60,000–65,000 plants per hectare. There was no irrigation during the crop growth period, and other management measures were consistent with local conventional production.

### 4.3. Soil Sampling and Measurement

Soil samples were collected after maize harvest in 2025. Soil samples of 0–20 cm and 20–40 cm soil layers were collected by cutting rings for the determination of soil BD, soil TP and soil three-phase ratio (solid–liquid–gas distribution) (with three replicates per plot). Meanwhile, additional soil samples were collected from these two soil layers and placed in hard plastic boxes; one additional soil sample was collected per plot and air-dried naturally in the laboratory for the analysis of soil aggregates.

The soil BD was measured by the cutting ring method. The soil three-phase ratio was determined using a soil three-phase analyzer (DIK-1150, Daiki, Konosu, Japan). The determination of soil aggregates was based on the method proposed by Elliott [46]. The soil aggregate analyzer (DIK-2012, Daiki, Konosu, Japan) was used for wet sieving to obtain soil water-stable aggregates of the following 6 particle sizes: >2 mm, 2–1 mm, 1–0.5 mm, 0.5–0.25 mm, 0.25–0.106 mm, and <0.106 mm, one replicate per sample for wet sieving. Maize yield was determined after harvest on sub-plots of 10 m^2^ with three replicates, and the actual crop yields were calculated based on a moisture content of 14%.

#### 4.3.1. Calculation Formula of Soil Physical Index

The calculation formulas for soil TP, GSSI and R are as follows [23,24]:

(1) TP=(1− BD∕2.65) × 100%

(2) $$GSSI={\left[\left(X_{g}-25\right)X_{y}X_{q}\right]}^{0.4769}$$

(3) $$R=\sqrt{{\left(X_{g}-50\right)}^{2}+{{\left(X_{y}-25\right)}^{2}+\left(X_{q}-25\right)}^{2}}$$

where X_g_ is the volume percentage of solid phase (%), X_y_ is the volume percentage of liquid phase (%), and X_q_ is the volume percentage of gas phase (%). Soil BD refers to the mass of soil per unit volume in its natural state. Soil TP refers to the percentage of the total pore volume in the overall soil volume. GSSI is a comprehensive indicator for quantitatively evaluating soil structural quality based on soil three-phase composition; the closer the GSSI is to 100, the closer the soil structure is to the ideal state. The R characterizes the deviation between the measured soil three-phase ratio and the ideal three-phase ratio (50:25:25); the higher the R, the more stable the soil structure.

#### 4.3.2. Calculation Formula of Soil Aggregate Index

The calculation formulas for the proportion of the *i*-th aggregate fraction (*W_i_*), MWD, GMD, and R_0.25_ are as follows [47]:

(4) $$W_{i}=\frac{W_{i}}{W_{t}}\times 100\%$$

(5) $$\mathrm{MWD}=\frac{\sum_{i=1}^{n}W_{i}{\bar{X}}_{i}}{\sum_{i=1}^{n}W_{i}}$$

(6) $$\mathrm{GMD}=\mathrm{EXP}\frac{\sum_{i=1}^{n}{W_{i}\ln\bar{X}}_{i}}{\sum_{i=1}^{n}W_{i}}$$

(7) $$R_{0.25}=\frac{m_{0.25}}{M}\times 100\%$$

where W*_i_* is the mass of the *i*-sized aggregate fraction (g), W*_t_* is the total mass of all aggregates (g), X*_i_* is the mean diameter of aggregates (mm), m_0.25_ is the mass of aggregates with soil particle size >0.25 mm (g), and M is the total mass of the tested sample (g).

### 4.4. Statistical Analysis

Data statistics were conducted using the software Excel 2016. Analysis of variance was conducted using SPSS 26.0. Normality and homogeneity of variance were tested prior to ANOVA. Significant differences were tested by Duncan’s multiple range test (*p* < 0.05). The Pearson method was used for correlation analysis. All figures were plotted using Origin 2024.

## 5. Conclusions

Soil amendments and bio-organic fertilizers significantly improved the physical properties and aggregate stability of albic soil and increased maize yield significantly. Compared with the control (T0), the application of soil amendment (T1) alone and its combination with bio-organic fertilizer (T2) optimized the soil three-phase structure of the 0–40 cm soil layer, especially the lower soil layer (20–40 cm), reduced R, increased GSSI and the content of >0.25 mm aggregates, and enhanced soil aggregate stability. There was a significant and positive correlation between maize yield and soil total porosity (TP), GSSI, and other indicators of size distribution and water stability of soil aggregates. Treatment of soil amendment and bio-organic fertilizer (T2) had the highest yield of maize. Therefore, soil physical properties are one of the key factors determining maize yield in albic soil areas.

Combined application technology of soil amendment and bio-organic fertilizer achieved multi-dimensional and full profile synergistic improvement of albic soil. The findings fill the research gap on layered regulation and collaborative improvement of albic soil structure, providing a theoretical basis and technical reference for ensuring food security of regional albic soil. We also recommend conducting long-term field studies on the effect of improvements on soil quality and crop productivity and verifying the applicability of technology over wider albic soil regions.

## Figures and Tables

**Table 1:** Soil three-phase ratio under different treatments.

Soil Layer (cm)	Treatment	Solid Phase (%)	Liquid Phase (%)	Gas Phase (%)
0–20	T0	49.10 ± 1.26 a	14.19 ± 0.85 b	36.71 ± 2.07 a
	T1	49.24 ± 1.45 a	16.84 ± 1.47 a	33.92 ± 0.35 b
	T2	48.75 ± 1.32 a	18.06 ± 0.89 a	33.19 ± 0.45 b
20–40	T0	64.83 ± 0.02 a	22.55 ± 0.43 b	12.62 ± 0.45 c
	T1	52.48 ± 2.97 b	21.84 ± 0.51 b	25.68 ± 2.45 a
	T2	54.98 ± 0.64 b	24.40 ± 0.19 a	20.62 ± 0.45 b

Note: different letters in the same column indicate significant differences between treatments (*p* < 0.05).

**Table 2:** Soil structure index and soil three-phase deviation value under different fertilization treatments.

Soil Layer (cm)	Treatment	Generalized Soil Structure Index	Soil Three-Phase Deviation Value
0–20	T0	89.98 ± 2.22 b	16.00 ± 2.11 a
	T1	94.25 ± 1.11 a	12.21 ± 0.90 b
	T2	95.58 ± 0.36 a	10.89 ± 0.22 b
20–40	T0	85.80 ± 0.65 b	19.48 ± 0.24 a
	T1	99.02 ± 0.78 a	4.88 ± 2.06 b
	T2	98.33 ± 0.38 a	6.66 ± 0.78 b

Note: different letters in the same column indicate significant differences between treatments (*p* < 0.05).

**Table 3:** Stability of soil aggregates under different treatments.

Soil Layer (cm)	Treatment	R_0.25_ (%)	MWD (mm)	GMD (mm)
0–20	T0	49.60 ± 0.02 b	0.56 ± 0.06 b	0.26 ± 0.02 b
	T1	56.40 ± 0.05 a	0.68 ± 0.06 a	0.32 ± 0.06 ab
	T2	61.11 ± 0.02 a	0.76 ± 0.04 a	0.39 ± 0.03 a
20–40	T0	41.06 ± 0.02 a	0.45 ± 0.02 b	0.20 ± 0.02 b
	T1	45.78 ± 0.07 a	0.58 ± 0.09 ab	0.24 ± 0.03 ab
	T2	51.71 ± 0.06 a	0.68 ± 0.07 a	0.31 ± 0.06 a

Note: different letters in the same column indicate significant differences between treatments (*p* < 0.05).

**Table 4:** Maize yield and yield increase rate under different treatments.

Treatment	Yield (kg·ha^−1^)	Increase (%) (Compared with T0)	Increase (%) (Compared with T1)
T0	9620.50 ± 166.30 b		
T1	10,914.30 ± 810.60 a	13.45	
T2	11,434.00 ± 483.00 a	18.85	4.76

Note: different letters in the same column indicate significant differences between treatments (*p* < 0.05).

**Table 5:** Application rate of fertilizers and amendment.

Treatment	Formula Fertilizer (kg·ha^−1^)	Soil Amendment (kg·ha^−1^)	Bio-Organic Fertilizer (kg·ha^−1^)
T0	600	0	0
T1	600	1500	0
T2	600	1500	2000

## Data Availability

The original contributions presented in this study are included in the article; further inquiries can be directed to the corresponding author.
